# Cluster analysis of autoencoder-extracted FDG PET/CT features identifies multiple myeloma patients with poor prognosis

**DOI:** 10.1038/s41598-023-34653-3

**Published:** 2023-05-15

**Authors:** Hyunjong Lee, Seung Hyup Hyun, Young Seok Cho, Seung Hwan Moon, Joon Young Choi, Kihyun Kim, Kyung-Han Lee

**Affiliations:** 1 Department of Nuclear Medicine, Samsung Medical Center, Sungkyunkwan University School of Medicine, 81 Irwon-Ro, Gangnam-Gu, Seoul, 06351 Republic of Korea; 2 Division of Hematology/Oncology, Department of Medicine, Samsung Medical Center, Sungkyunkwan University School of Medicine, 81 Irwon-Ro, Gangnam-Gu, Seoul, 06351 Republic of Korea

**Keywords:** Oncology, Cancer imaging, Haematological cancer

## Abstract

F-18 fluorodeoxyglucose positron emission tomography/computed tomography (FDG PET/CT) is a robust imaging modality used for staging multiple myeloma (MM) and assessing treatment responses. Herein, we extracted features from the FDG PET/CT images of MM patients using an artificial intelligence autoencoder algorithm that constructs a compressed representation of input data. We then evaluated the prognostic value of the image-feature clusters thus extracted. Conventional image parameters including metabolic tumor volume (MTV) were measured on volumes-of-interests (VOIs) covering only the bones. Features were extracted with the autoencoder algorithm on bone-covering VOIs. Supervised and unsupervised clustering were performed on image features. Survival analyses for progression-free survival (PFS) were performed for conventional parameters and clusters. In result, supervised and unsupervised clustering of the image features grouped the subjects into three clusters (A, B, and C). In multivariable Cox regression analysis, unsupervised cluster C, supervised cluster C, and high MTV were significant independent predictors of worse PFS. Supervised and unsupervised cluster analyses of image features extracted from FDG PET/CT scans of MM patients by an autoencoder allowed significant and independent prediction of worse PFS. Therefore, artificial intelligence algorithm–based cluster analyses of FDG PET/CT images could be useful for MM risk stratification.

## Introduction

Multiple myeloma (MM) is the second most common hematologic malignancy in adults^1^. The diagnosis of this plasma cell neoplasm requires greater than 10% clonal bone marrow plasma cells or biopsy-proven plasmacytoma and the presence of myeloma-defining events such as hypercalcemia and anemia^2^. Patients are treated with a multi-drug regimen, followed in eligible cases by autologous stem cell transplantation (ASCT)^3^. Newly diagnosed patients have a median survival of approximately 10 years, but relapse occurs in about 40% of cases^4^. Noninvasive methods to stratify patients with high risk could help to optimize MM management.

F-18 fluorodeoxyglucose positron emission tomography/computed tomography (F-18 FDG PET/CT) can detect medullary and extramedullary disease (EMD) in the whole body, and it is recommended for the initial diagnostic workup and follow-up of patients with MM^3^. Beyond their diagnostic usefulness, FDG PET/CT findings also have prognostic value. In MM, the presence of EMD and high standardized uptake value (SUV) are reported to be independent indicators of poor progression-free survival (PFS)^5^. Volumetric parameters such as metabolic tumor volume (MTV) and total lesion glycolysis (TLG) can provide additional prognostic information^6,7^.

Recently, interest in artificial intelligence algorithms that can extract prognostic indicators from imaging studies of cancer patients has been rising. Among various algorithms, autoencoders are widely utilized to perform unsupervised clustering of unlabeled radiologic and nuclear medicine image data. An autoencoder has two parts, an encoder that compresses the input data with dimension reduction or denoising and extracts essential features from high-dimensional image data and a decoder that regenerates the input data from the encoded data^8^. A previous study reported an autoencoder that offered a fair diagnostic performance for lung nodule classification^9^. Another study showed the usefulness of an autoencoder for predicting metabolic changes in FDG PET of the brain^10^. However, the ability of an autoencoder to analyze FDG PET/CT images of MM patients has not been previously explored.

In this work, we investigated the prognostic value of image features extracted from FDG PET/CT scans of MM patients by a convolutional autoencoder. Supervised and unsupervised clustering were tested on the extracted PET/CT features. The prognostic value of the extracted feature clusters was then compared with that of MTV measurements.

## Methods

### Subjects and clinical data

**Figure 1:**
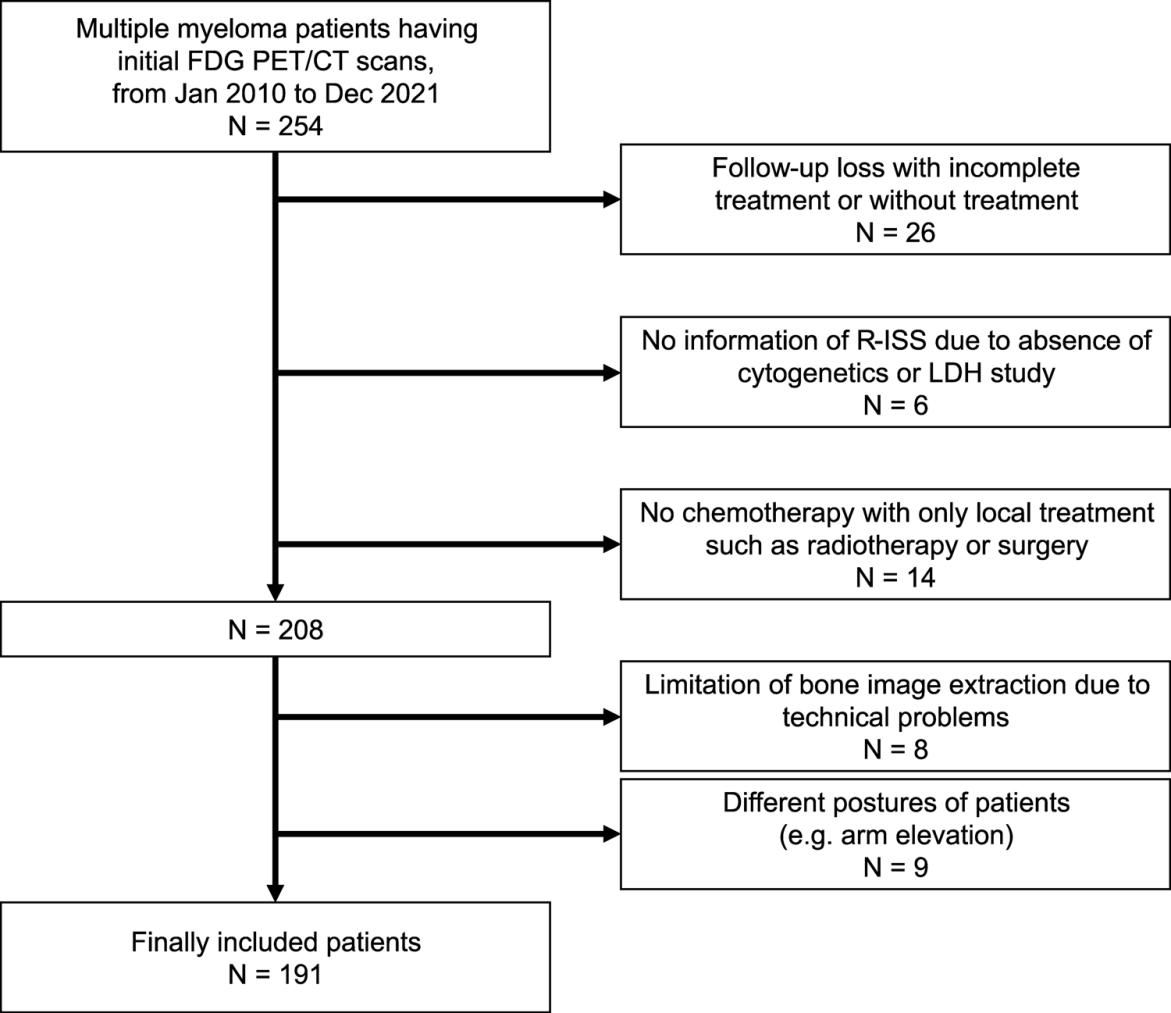
Scheme for selecting study subjects. Candidates retrospectively enrolled were 254 multiple myeloma patients who underwent FDG PET/CT for initial staging. Among them, 26 cases were excluded due to follow-up loss without completion of treatment, and 14 were excluded because they received only local treatment. An additional 6 cases were excluded from analysis because information on the R-ISS stage was unavailable, and 17 cases were excluded due to technical problems. Therefore, 191 patients were included as study subjects for analysis. *R-ISS* revised multiple myeloma international staging system.

The study candidates were 254 MM patients who underwent FDG PET/CT as an initial workup at our institution between January 2010 and December 2021. Among the candidates, 26 patients were excluded due to follow-up loss without treatment completion. Fourteen patients who received only surgery or local radiotherapy were also excluded. Six patients were excluded due to the absence of cytogenetic studies or serum lactate dehydrogenase (LDH) measurements. An additional 17 cases were excluded due to misregistration between the PET and CT or imaging in different postures (arm elevation). Therefore, 191 patients were included in this study (Fig. 1). Samsung Medical Center institutional review board approved this retrospective study (IRB #2022-10-106). Written informed consent was waived by Samsung Medical Center institutional review board due to the retrospective design of this study. All methods were carried out in accordance with relevant guidelines and regulations.

All clinical information and laboratory results were acquired from electronic medical records. As clinical information, we collected sex, age, date of diagnosis, chemotherapeutic regimen, ASCT history, date of relapse or progression, and date of death or last follow-up. We also collected the following laboratory results: serum albumin, serum β2 microglobulin, serum LDH, serum calcium, serum creatinine, hemoglobin, and cytogenetic study results. The Revised Multiple Myeloma International Staging System (R-ISS) stage of each patient was determined using the laboratory test results^11^.

### FDG PET/CT acquisition

All patients fasted for at least six hours and had blood glucose levels of less than 200 mg/dL at the time of PET/CT imaging. Whole-body PET and CT images were acquired 60 min after an injection of 5.0 MBq/kg FDG (without intravenous or oral contrast) on a Discovery LS, a Discovery STE, or a Discovery MI DR PET/CT scanner (GE Healthcare, Milwaukee, WI, USA). Continuous spiral CT was performed with an 8-slice helical CT (140 keV, 40–120 mA, Discovery LS) or 16-slice helical CT (140 keV, 30–170 mA, Discovery STE; 120 keV, 30–100 mA, Discovery MI DR). An emission scan was then obtained from head to thigh for 4 min per frame in 2-dimensional mode (Discovery LS), 2.5 min per frame in 3-dimensional mode (Discovery STE), or 2 min per frame in 3-dimensional mode (Discovery MI DR). PET images were reconstructed using CT for attenuation correction with the ordered-subsets expectation maximization algorithm with 28 subsets and 2 iterations (matrix 128 × 128, voxel size 4.3 × 4.3 × 3.9 mm, Discovery LS), ordered-subsets expectation maximization algorithm with 20 subsets and 2 iterations (matrix 128 × 128, voxel size 3.9 × 3.9 × 3.3 mm, Discovery STE), or ordered-subsets expectation maximization algorithm with 18 subsets and 4 iterations (matrix 192 × 192, voxel size 3.9 × 3.9 × 3.3 mm, Discovery MI DR). The SUV was calculated by adjusting for the administered FDG dose and patient body weight.

### FDG PET/CT image analysis

The image analysis scheme is illustrated in Supplementary Fig. 1. FDG PET/CT images were retrieved to MIM Encore version 7.0 (MIM Software Inc., Cleveland, OH). On the CT images, a volume-of-interest (VOI) with Hounsfield units (HU) above 150 was drawn from the skull base to the upper thigh. This process resulted in inclusion of high-attenuation structures such as calcification or foreign materials and exclusion of some osteolytic lesions with HU below 150. Therefore, an experienced nuclear medicine physician carefully reviewed and manually corrected all the VOIs to include the entire skeleton and exclude non-skeletal high-attenuation structures. The VOIs and PET images were saved in DICOM format. From the VOIs, conventional maximum SUV (SUVmax) and mean SUV (SUVmean) values were obtained. The volumetric metabolic parameters of MTV and TLG were measured using an SUV of 2.5 as the threshold.

### Feature extraction

The VOIs and PET images were loaded into Python version 3.9 via the *pydicom* and *rt_utils* libraries. Voxels covering only bones were extracted using a previously drawn VOI as a mask. Anterior view maximum intensity projection (MIP) images that were uniformly resized into 64-pixel widths and 128-pixel heights were then constructed and used as input data for the convolutional autoencoder that performed the feature extraction.

The encoder part of the convolutional autoencoder was composed of 3 convolutional layers and 3 max-pooling layers. The sizes of all the convolutional filters were set to 3 × 3, and the filter numbers were set to 16, 20, and 24. Max-pooling was applied on each 2 × 2 region. The decoder part was composed of 4 convolutional layers and 3 up-sampling layers. The sizes of all the convolutional filters were set to 3 × 3, and the filter numbers set to 24, 20, 16, and 1. Up-sampling was applied on each 2 × 2 region. The *ReLu* function was used as the activation function in all the encoder layers and the first three decoder layers. The *Sigmoid* function was used as the activation function in the last decoder layer. The input data were divided into training and test sets with a 1:1 ratio. An Adam optimizer was adopted, and the learning rate was 0.01. The convolutional autoencoder was trained with four batch sizes and 100 epochs. The trained convolutional autoencoder extracted 3072 features from each MIP image.

Those 3072 extracted features were then subjected to supervised and unsupervised clustering. For the unsupervised clustering, K-means clustering was performed in Python version 3.9. The number of clusters was set at three, and the maximum iteration was set at 300. For the supervised clustering, the *survClust* package in R software was used^12^. It provides an outcome-weighted clustering algorithm for survival stratification of multi-dimension data. To avoid overfitting, the supervised clustering was performed in 10 rounds of threefold cross validation to classify the subjects into three clusters via the *cv.survclust* function. A principal component analysis (PCA) was performed to visualize the clustering results using the *PCA* function in the *factoextra* package for R software.

### Statistical methods and survival analysis

Shapiro–Wilk tests were performed to evaluate the normality of the conventional FDG parameters: SUVmax, SUVmean, MTV, and TLG. Kruskal–Wallis tests were used to compare those parameters among the different cluster groups. Two-sided P values < 0.05 were deemed statistically significant. All statistical analyses were performed using R software (v. 4.0.4, https://www.R-project.org/, R Foundation for Statistical Computing, Vienna, Austria)^13^.

PFS was the endpoint of our survival analysis. Events were MM relapse, progression, or any-cause death. Recurrence and progression were based on bone marrow examinations or the results of serial laboratory tests. PFS was defined as the duration from the date of initial diagnosis to the date of an event or last follow-up**.** The median follow-up was 665 days (50–3272 days). An observation was considered right censored and analyzed accordingly if the subject did not have an event at the end of follow-up or was lost to follow-up.

Age was divided into three ranges by tertiles with cutoff points at 58 and 67 years. MTV was categorized as low, moderate, and high based on cutoff values that best discriminated PFS when explored by the *surv_cutpoint* function in the *survminer* package of R software. MTV was also divided into three ranges with cutoffs of 22.0 and 83.6 cm^3^.

Kaplan–Meier analysis with log-rank tests and univariate Cox proportional hazards models with hazard ratios (HRs) were used to evaluate the prognostic power of the major variables: sex, age, R-ISS stage, chemotherapy regimen, radiotherapy, ASCT, hypercalcemia, renal insufficiency, anemia, and the presence of bone lesions or EMD. Unsupervised cluster, supervised cluster, and MTV were also included in the survival analysis. Furthermore, multivariate Cox analysis was performed using variables with log-rank test P values < 0.05 in the univariate analysis. Because of potential multicollinearity among the unsupervised cluster, supervised cluster, and MTV groups, the multivariate survival analysis was repeated while including just one of those three variables at a time.

## Results

### Demographic data

The clinical characteristics and demographics of the study subjects are described in Table 1. The median age was 62 years (range: 41–90 years), and 53.9% were male. The subjects were most often R-ISS stage II (52.4%), and VTD (bortezomib, thalidomide, and dexamethasone) was the most common chemotherapeutic regimen (43.5%). Radiotherapy was performed in 9.9% of the patients, and 52.9% of subjects underwent ASCT. The median MTV on FDG PET/CT was 33.1 cm^3^ (range: 0–1452.4 cm^3^).

**Table 1:** Demographic and clinical characteristics of the multiple myeloma patients.

Characteristics	Patients, n (%)
Sex, male	103 (53.9)
Age, years	
< 58	68 (35.6)
58–67	66 (34.6)
67 ≤	57 (29.8)
R-ISS stage	
I	51 (26.7)
II	100 (52.4)
III	40 (20.9)
Chemotherapy regimen	
VTD	83 (43.5)
VMP	49 (25.7)
Other	59 (30.9)
Radiotherapy	19 (9.9)
Autologous stem cell transplantation	101 (52.9)
Hypercalcemia	25 (13.1)
Renal insufficiency	46 (24.1)
Anemia	96 (50.3)
Bone lesions	165 (86.4)
Extramedullary disease	35 (18.3)
Metabolic tumor volume (MTV)	
< 22.0 cm^3^ (low)	87 (45.5)
22.0–83.6 cm^3^ (moderate)	48 (25.1)
83.6 cm^3^ ≤ (high)	56 (29.3)
Unsupervised clustering	
A	87 (45.5)
B	81 (42.4)
C	23 (12.0)
Supervised clustering	
A	94 (49.2)
B	68 (35.6)
C	29 (15.2)
Instrument	
Discovery LS	28 (14.7)
Discovery STE	132 (69.1)
Discovery MI DR	31 (16.2)

*VTD* bortezomib, thalidomide, dexamethasone, *VMP* bortezomib, melphalan, prednisone, *R-ISS* revised multiple myeloma international staging system.

### Clustering based on extracted features

**Figure 2:**
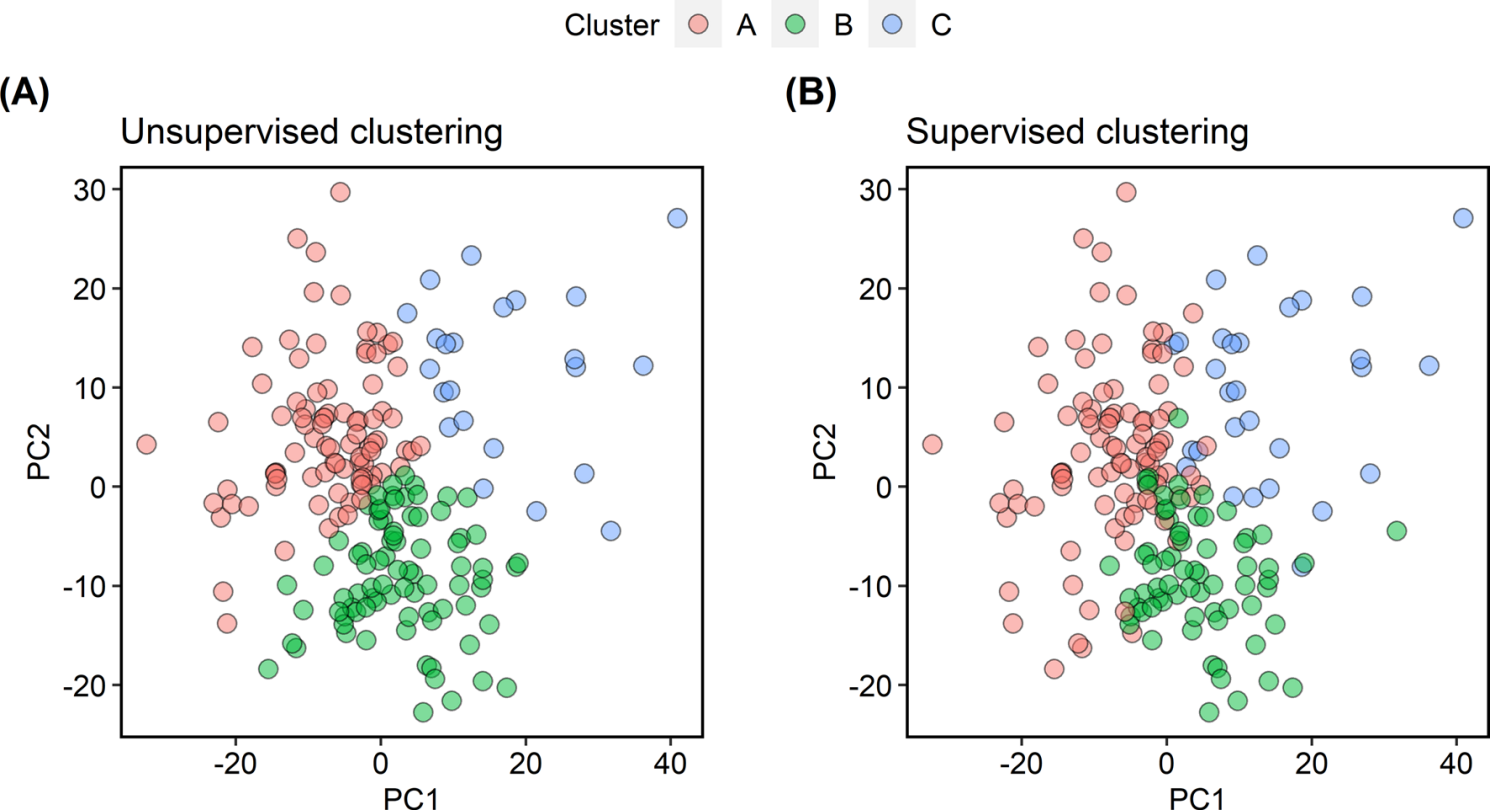
PCA plots displaying the results of the cluster analyses. Subjects were grouped into three clusters via unsupervised (**A**) and supervised clustering (**B**). The clustering patterns were similar between the two cluster analysis methods. *PCA* principal component analysis.

**Figure 3:**
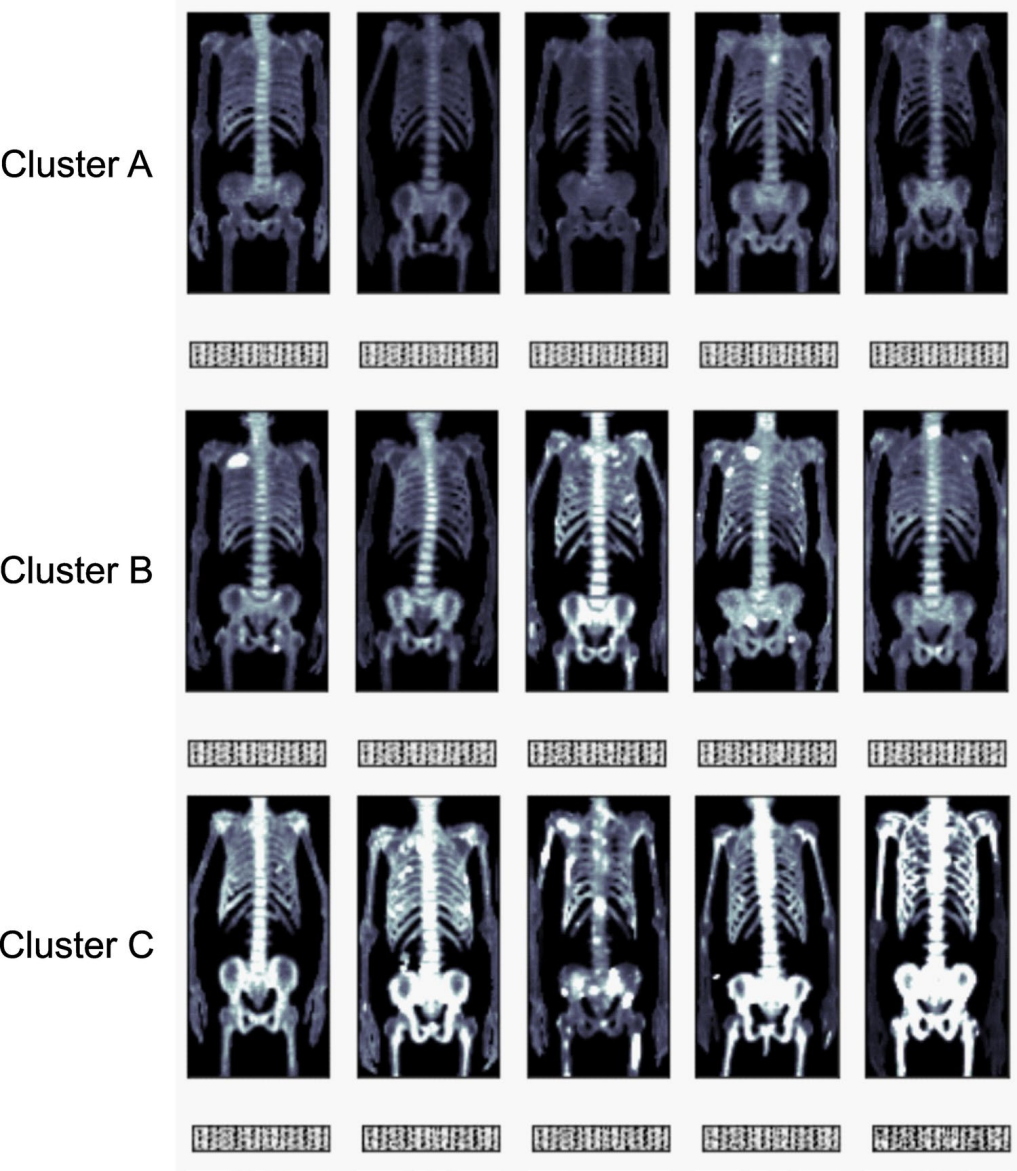
Representative anterior view MIP images of cases belonging to each cluster. Five representative cases are displayed for each cluster. The upper panels of each row are the MIP images of FDG PET/CT scans. The lower panels of each row are the encoded images showing the extracted features. There are visually apparent increases in FDG uptake intensity from clusters A to B and from B to C. *MIP* maximum intensity projection.

**Figure 4:**
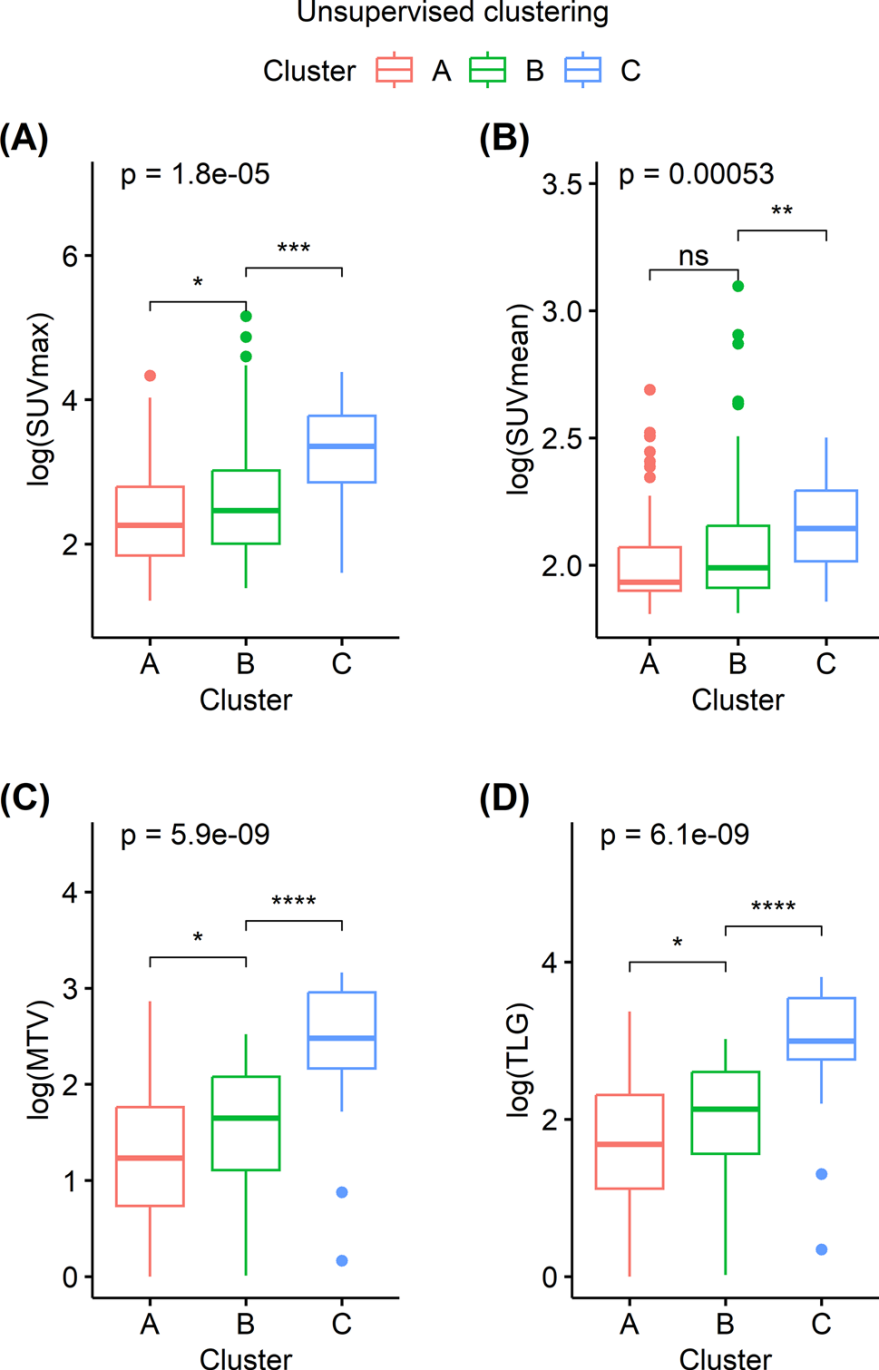
Comparison of conventional FDG parameters according to the clusters obtained by the unsupervised analysis. The logarithms of SUVmax, SUVmean, MTV, and TLG differed significantly between cluster groups (all P < 0.001). All parameters were significantly greater in patients in cluster C than in the other clusters (all P < 0.001, except for SUVmean [P ≤ 0.01]). In addition, SUVmax, MTV, and TLG were significantly greater in patients in cluster B than in cluster A (P ≤ 0.05). *ns* not significant; *P ≤ 0.05; **P ≤ 0.01; ***P ≤ 0.001; ****P ≤ 0.0001. SUVmax, maximum standardized uptake value; MTV, metabolic tumor volume; TLG, total lesion glycolysis; SUVmean, mean standardized uptake value.

**Figure 5:**
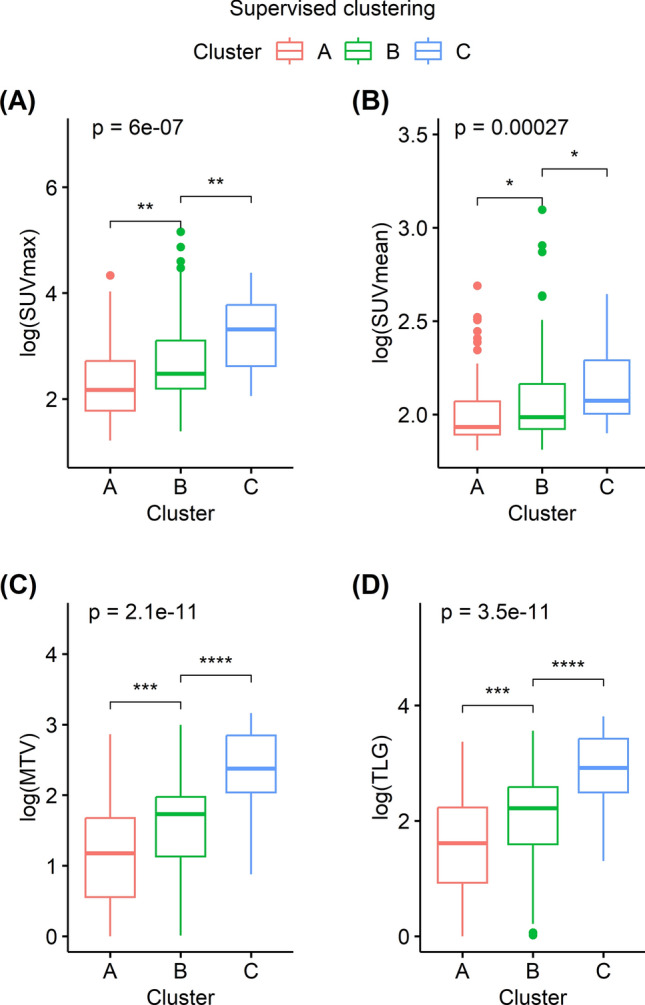
Comparison of conventional FDG parameters according to clusters obtained by the supervised analysis. The logarithms of SUVmax, SUVmean, MTV, and TLG differed significantly between cluster groups (all P < 0.001). All parameters were significantly greater in patients in cluster C than in the other clusters, and in patients in cluster B compared with those in cluster A. *ns* not significant; *P ≤ 0.05; **P ≤ 0.01; ***P ≤ 0.001; ****P ≤ 0.0001. *SUVmax*, maximum standardized uptake value, *MTV* metabolic tumor volume, *TLG* total lesion glycolysis, *SUVmean* mean standardized uptake value.

Supervised and unsupervised clustering were performed on the 3072 features extracted by the convolutional autoencoder. The pattern of clustering produced by the supervised and unsupervised methods was similar. Figure 2 illustrates the clusters as PCA plots. Both the supervised and unsupervised clustering sorted 75, 63, and 21 subjects into clusters A, B, and C, respectively. Representative FDG PET MIP images of five subjects in each cluster are displayed in Fig. 3. Those subjects were sorted into the same clusters by both the supervised and unsupervised methods. The subjects in clusters A, B, and C displayed an increasing extent of high FDG uptake in that order. Thus, SUVmax, SUVmean, MTV, and TLG were significantly higher for subjects in cluster C than in those of the other clusters following both supervised and unsupervised clustering (Figs. 4 and 5, all *P* < 0.001).

### Prognostic validation of clustering

**Figure 6:**
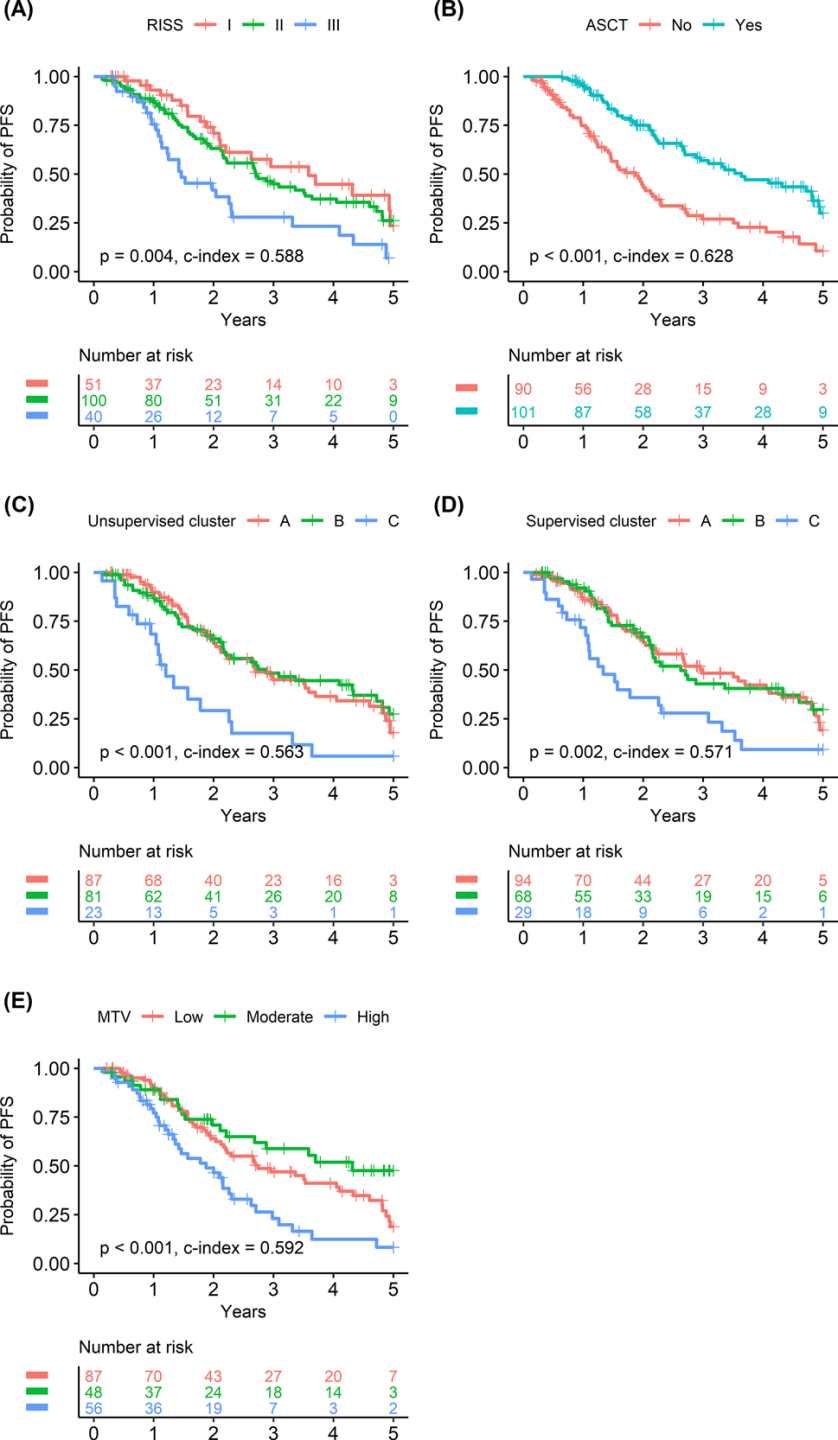
Kaplan–Meier survival curves showing the probability of PFS according to the major variables. R-ISS stage (**A**) and ASCT history (**B**) were clinical prognostic factors that significantly discriminated a high risk of poor PFS. Unsupervised cluster C (**C**), supervised cluster C (**D**), and high MTV (**E**) were FDG PET variables associated with significantly worse PFS. *R-ISS* revised multiple myeloma international staging system, *ASCT* autologous stem cell transplantation, *PFS* progression-free survival, *MTV* metabolic tumor volume.

Kaplan–Meier survival analysis demonstrated that R-ISS stage, ASCT, unsupervised clusters, supervised clusters, and MTV were significant predictors of PFS (Fig. 6). Specifically, R-ISS stage III was associated with worse PFS than stage I (*P* = 0.003) and II (*P* = 0.009), but there was no significant difference between R-ISS stages I and II. Similarly, both supervised and unsupervised cluster C were associated with worse PFS than clusters A and B, but there was no significant difference between clusters A and B. High MTV was associated with worse PFS than low or moderate MTV (both *P* < 0.001), with no significant difference between low and moderate MTV. Univariate Cox regression analysis showed that age, R-ISS stage, anemia, chemotherapy regimen, ASCT, unsupervised cluster, supervised cluster, and MTV were significant prognostic factors for PFS (Table 2).

**Table 2:** Univariate Cox regression analysis for PFS in all subjects.

Variable	Categories	Hazard ratio	95% CI	*P* value	Log-rank test
Sex	Female				0.113
	Male	1.365	0.927–2.011	0.115	
Age	< 58				0.006
	58–67	1.334	0.829–2.147	0.235	
	67 ≤	2.126	1.319–3.429	0.002	
Chemotherapy regimen	VTD				< 0.001
	VMP	2.472	1.544–3.958	< 0.001	
	Other	1.650	1.021–2.668	0.041	
Radiotherapy	No				0.624
	Yes	0.844	0.426–1.671	0.626	
Autologous stem cell transplantation	No				< 0.001
	Yes	0.426	0.289–0.626	< 0.001	
R-ISS stage	I				0.004
	II	1.250	0.758–2.062	0.382	
	III	2.375	1.345–4.193	0.003	
Hypercalcemia	No				0.712
	Yes	1.111	0.633–1.951	0.714	
Renal insufficiency	No				0.090
	Yes	1.457	0.947–2.243	0.087	
Anemia	No				0.008
	Yes	1.684	1.143–2.482	0.008	
Bone lesions	No				0.990
	Yes	1.003	0.581–1.733	0.990	
Extramedullary disease	No				0.545
	Yes	0.852	0.506–1.432	0.544	
Unsupervised cluster	A				< 0.001
	B	0.931	0.612–1.416	0.739	
	C	2.694	1.556–4.662	< 0.001	
Supervised cluster	A				0.002
	B	0.958	0.612–1.485	0.848	
	C	2.234	1.359–3.676	0.001	
MTV	Low				< 0.001
	Moderate	0.660	0.389–1.120	0.124	
	High	1.865	1.212–2.869	0.005	

*PFS* progression-free survival, *VMP* bortezomib, melphalan, and prednisone, *R-ISS*, revised multiple myeloma international staging system, *MTV* metabolic tumor volume, *CI* confidence interval, *VTD* bortezomib, thalidomide, dexamethasone.

Multivariate Cox regression analysis was performed with those significant univariate prognostic factors but including only one of supervised cluster, unsupervised cluster, or MTV at a time (Table 3). Due to multicollinearity with ASCT history, chemotherapy regimen was not included in the analysis. Those results reveal that ASCT was a significant independent prognostic factor in all instances (all *P* < 0.001). In addition, unsupervised cluster C (HR = 2.68; *P* < 0.001), supervised cluster C (HR = 2.42; *P* = 0.002), and MTV (HR = 2.01; *P* < 0.001) were independent prognostic factors when they were individually included in the analysis (Table 3).

**Table 3:** Multivariate Cox regression analysis for PFS in all subjects.

Variable		Unsupervised cluster			Supervised cluster			MTV		
		HR	95% CI	P	HR	95% CI	P	HR	95% CI	P
Age	< 58									
	58 ~ 67	0.97	0.59–1.59	0.897	0.92	0.55–1.51	0.730	0.97	0.87–1.61	0.916
	67 ≤	0.84	0.43–1.61	0.591	0.78	0.41–1.51	0.468	0.81	0.42–1.59	0.540
ASCT	No									
	Yes	0.37	0.21–0.63	< 0.001	0.33	0.19–0.57	< 0.001	0.38	0.22–0.66	< 0.001
R-ISS stage	I									
	II	0.89	0.53–1.50	0.663	0.84	0.50–1.43	0.523	0.85	0.50–1.45	0.549
	III	1.28	0.68–2.42	0.444	1.17	0.61–2.26	0.632	1.42	0.75–2.71	0.286
Anemia	No									
	Yes	1.40	0.92–2.12	0.117	1.33	0.86–2.04	0.196	1.30	0.85–2.00	0.230
Unsupervised cluster	A									
	B	1.05	0.69–1.61	0.822						
	C	2.68	1.50–4.79	< 0.001						
Supervised cluster	A									
	B				0.98	0.63–1.54	0.940			
	C				2.42	1.37–4.29	0.002			
MTV	Low									
	Moderate							0.80	0.46–1.38	0.419
	High							2.01	1.28–3.16	0.002

*ASCT* autologous stem cell transplantation, *PFS* progression-free survival, *R-ISS* revised multiple myeloma international staging system, *MTV* metabolic tumor volume.

Given the strong prognostic association of ASCT, we performed an additional survival analysis in subgroups according to this variable. In subjects who had not received ASCT, the Kaplan–Meier analysis demonstrated that unsupervised cluster C, supervised cluster C, and high MTV were significant predictors of worse PFS (Supplementary Fig. 2). In that subgroup, Cox regression analysis showed that R-ISS stage, unsupervised cluster, supervised cluster, and MTV were significant univariate prognostic factors for PFS (Supplementary Table 1). Multivariate Cox regression analysis demonstrated that unsupervised cluster C (HR = 3.36), supervised cluster C (HR = 4.07), and MTV (HR = 2.83) were independent prognostic factors (Supplementary Table 2).

In contrast, none of those variables was significantly associated with PFS in subjects who received ASCT (Supplementary Fig. 2). 

## Discussion

In this study, we have shown that autoencoders can successfully extract image features from FDG PET/CT scans of MM patients. The supervised and unsupervised cluster groups contained different patterns of bone metabolic activity as demonstrated by SUV, MTV, and TLG measurements. The Kaplan–Meier survival analysis and Cox regression models revealed that both the supervised and unsupervised clusters were significantly associated with patient outcomes.

FDG PET/CT provides high-contrast whole-body images of glucose metabolism and is a robust diagnostic modality for staging and treatment response assessment in various malignant diseases^14^. In patients with MM, FDG PET/CT has higher sensitivity for bone lesion detection than conventional radiography and offers findings concordant with magnetic resonance imaging (MRI)^15,16^. Moreover, the bone lesions and EMD detected by this modality are a prognostic predictor of survival outcomes^5,17^. Previous studies have indicated that estimations of tumor burden made from FDG PET/CT images can be used for risk stratification. Thus, MTV and TLG, which represent the tumor burden, can provide particularly useful prognostic information about MM patients^6,18^.

Selecting the bone on FDG PET/CT images to measure the tumor burden in the skeletal system can be achieved automatically and precisely with the aid of artificial intelligence (AI). We are aware of only two previous studies that investigated the application of AI to PET/CT analyses in MM patients. One was the study of Xu et al. who used AI to detect bone lesions on Ga-68 Pentixafor PET/CT images^19^. The other was by Mesguich and coworkers, who used machine learning during model construction to diagnose diffuse infiltrative disease with radiomic FDG PET/CT parameters. However, their study did not use AI for feature extraction^20^. To the best of our knowledge, this study is the first to use AI to extract image features from FDG PET/CT scans of MM patients.

In this study, we successfully extracted image features from FDG PET/CT scans using a convolutional autoencoder. Convolutional autoencoders have several advantages for analyzing medical images such as CT, MRI, and FDG PET/CT that generate large data files with many pixels and thus require data compression of essential features for further analysis. Radiomic features are generally an excellent way to represent and characterize the patterns in medical images^21^. However, it is difficult to compress and reproduce input data via hand-crafted radiomic parameters such as texture parameters^22^. In this study, an AI algorithm was utilized to extract image feature with superior reproducibility and low redundancy. Among different feature extraction AI algorithms, convolutional autoencoders have been shown to provide better classification accuracy compared with other methods^23^ and were thus employed in our study.

Unsupervised clustering of FDG PET image features with k-means algorithms was confirmed to be a straightforward method that could be routinely applied in clinics. In this study, we also tested supervised clustering with the *survClust* algorithm and successfully provided outcome-weighted clustering of survival data^12^. The supervised algorithm weighs each feature by incorporating a vector of the Cox regression hazard ratio. Clustering membership was explored by integrating patient outcome–weighted distance matrices, and cross-validation was performed to achieve more reproducible clustering results without overfitting. Although this algorithm was originally introduced to analyze genomics data and find prognostic molecular subtypes, imaging data and genomics data share the common aspect of high-dimensionality, so image data can also be analyzed with this algorithm. To our knowledge, this study is the first actual attempt to use survival outcome–weighted supervised clustering with imaging data. Our results show that the supervised and unsupervised clustering produced highly similar grouping patterns in both the PCA plot and survival analysis results. At the same time, our results indicate that supervised clustering can be extended to imaging data. Compared with genomics data, applying clustering to imaging features could be considered limited by the low explainability of black-box AI algorithms. In that regard, supervised clustering for radiomic data could have the advantage of better interpretability than the unlabeled features extracted by unsupervised AI algorithms.

In our results, patients belonging to cluster C had significantly worse PFS than those in the other clusters. This is consistent with the high tumor burden in the FDG PET/CT images of the patients in cluster C, which is known to be a poor prognostic factor in MM. Interestingly, the patients in cluster B showed a moderate level of tumor burden in their bones, but they did not have worse PFS than those in cluster A, who had the lowest skeletal tumor burden.

MM patients with high MTV on FDG PET/CT are recognized as having a high risk of disease progression^6,18^. Our study also demonstrates significantly worse outcomes in patients with an MTV > 83.6 cm^3^. This is not remarkably different from the previously proposed optimum MTV cutoffs of 42.2 and 56.4 cm^3,6,18^. In our study, patients with moderate MTV did not show significantly worse PFS compared to those with low MTV, and even appeared to have a tendency for better long-term prognosis. Although the precise reason for this is not clear, it might suggest that MTV alone has limited accuracy for stratifying long-term risk of relapse unless tumor burden is sufficiently high.

In addition to image feature clusters and MTV, we have demonstrated a strong association between worse PFS and patients who did not receive ASCT, which is included in the standard of care for young patients with newly diagnosed MM. This is consistent with the finding of previous studies^24,25^. In that subgroup of patients, cluster C and high MTV remained significant independent predictors of poor outcomes. These results imply that for MM patients who do not receive ASCT, a high tumor burden with cluster C features and high MTV indicate a high risk of disease progression or relapse, so those patients might require follow-up at shorter-than-average intervals.

The PCA plots do not display obviously distinct visual patterns between cluster groups. The MIP images of subjects in each cluster demonstrate a greater extent of high FDG uptake, but they do not show other cluster group–specific image patterns. Thus, the AI algorithms might be limited in discerning differences in image patterns, other than the extent of high FDG uptake, that characterize MM patients at high risk.

In this study, we also tried repeating our clustering analysis using images showing only the bones with SUV above 2.5 as input data to test the effect of excluding bone marrow with low FDG uptake from the input data. This, however, did not significantly influence the clustering results. Further developments in AI algorithm methods are therefore warranted to help circumvent this issue in analyzing FDG PET/CT images from MM patients. Nevertheless, our method was sufficient to discern cluster C from clusters A and B despite the presence of gray zones on the PCA plots. Furthermore, the cluster analysis classification demonstrated good power for predicting PFS, supporting its potential usefulness for stratifying high-risk MM patients.

Although MTV demonstrated prognostic value that was comparable to that provided by cluster analysis, there are advantages to using the clustering analysis with autoencoder-extracted features. First, whereas MTV measurement depends on selection of an optimum SUV threshold, the clustering analysis is performed on the whole skeleton with a constant anatomical reference. Second, the autoencoder-extracted features potentially contain greater abundance of information about image features that are not provided by simple MTV measurements.

This study has several limitations. One is that 2-dimensional anterior view MIP images were used as autoencoder input, rather than 3-dimensional image data, whose massive size is difficult to handle. Further investigations of clustering analyses of 3-dimensional image data will thus strengthen our findings in this study. In addition, PET images limited to the torso were used. However, most osseous MM lesions are located between the skull base and upper thigh. Finally, clustering was performed with images of only the bones and did not include EMD lesions, but our results show that the presence of EMD was not associated with PFS.

In conclusion, the FDG PET/CT image features of MM patients were successfully extracted by a convolutional autoencoder. Both supervised and unsupervised clustering of the extracted features allowed significant and independent prediction of worse PFS. Although remarkable superiority over MTV was not demonstrated, our results support the usefulness of AI algorithm–based cluster analyses of FDG PET/CT images for risk stratification of patients with MM.

## Supplementary Information

Supplementary Figures.

Supplementary Tables.

## Data Availability

The data generated in this study are available upon request from the corresponding author.
