# Supplementary material for: Cluster analysis of autoencoder-extracted FDG PET/CT features identifies multiple myeloma patients with poor prognosis
Source: Sci Rep. 2023 May 15;13:7881. doi: 10.1038/s41598-023-34653-3 (PMC10185699; doi:10.1038/s41598-023-34653-3)
Supplement: Supplementary file 1 — Supplementary Figures. [file 41598_2023_34653_MOESM1_ESM.docx]

**
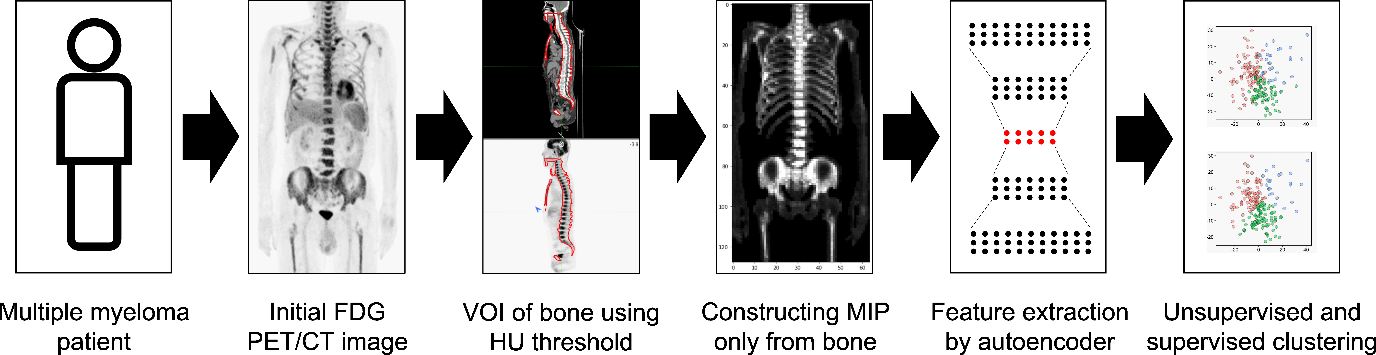
SUPPLEMENTARY FIGURES**

Supplementary Figure 1. Study scheme

Initial FDG PET/CT images of multiple myeloma patients were retrieved to MIM Encore version 7.0 (MIM Software Inc., Cleveland, OH). On CT image, volume-of-interests with Hounsfield units above 150 were drawn from skull base to upper thigh. Subsequently, maximum intensity projection images with anterior view were constructed by voxels covering only bones. Image features were extracted by convolutional autoencoder. Finally, unsupervised and supervised clustering were conducted for extracted 3,072 features.


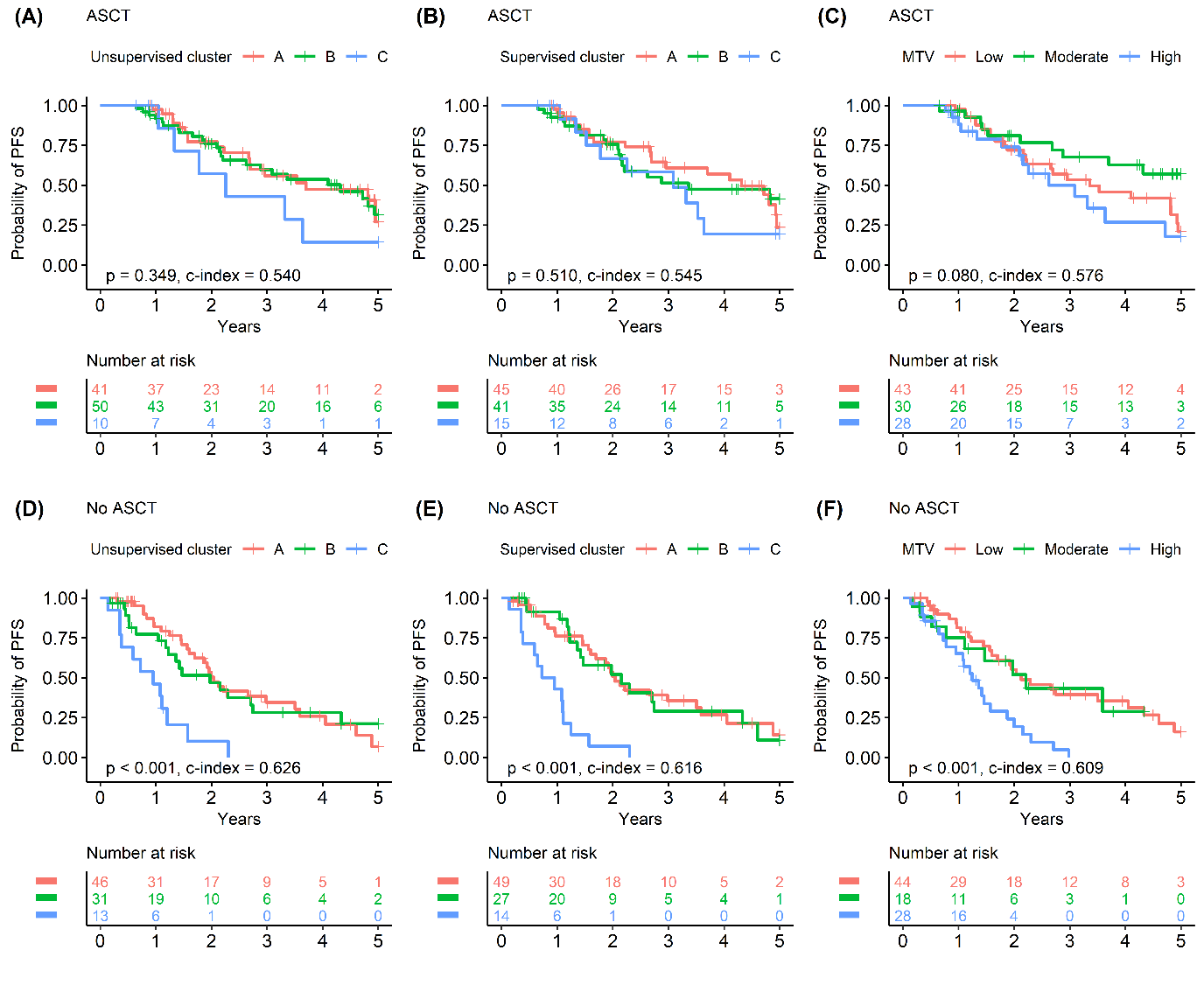
Supplementary Figure 2. Survival curves according to the significant prognostic factors in subgroup analyses

In subjects with ASCT, unsupervised clustering (A), supervised clustering (B), and MTV (C) could not discriminate high risk group for PFS significantly. On the contrary, unsupervised cluster C (D), supervised cluster C (E), and high MTV group (F) showed significantly worse prognosis for PFS than other clusters or groups in subjects without ASCT.

ASCT, autologous stem cell transplantation; MTV, metabolic tumor volume; PFS, progression-free survival
