# Supplementary material for: Cluster analysis of autoencoder-extracted FDG PET/CT features identifies multiple myeloma patients with poor prognosis
Source: Sci Rep. 2023 May 15;13:7881. doi: 10.1038/s41598-023-34653-3 (PMC10185699; doi:10.1038/s41598-023-34653-3)
Supplement: Supplementary file 2 — Supplementary Tables. [file 41598_2023_34653_MOESM2_ESM.docx]

**SUPPLEMENTARY TABLES**

| **Variable** | **Categories** | **Hazard ratio** | **95% confidence interval** | **P** | **P of log-rank test** |
| --- | --- | --- | --- | --- | --- |
| Sex | Female |  |  |  | 0.471 |
|  | Male | 1.215 | 0.712-2.073 | 0.475 |  |
| Age | < 58 |  |  |  | 0.617 |
|  | 58~67 | 0.645 | 0.248-1.681 | 0.370 |  |
|  | 67 ≤ | 0.656 | 0.274-1.571 | 0.344 |  |
| Chemotherapy regimen | VTD |  |  |  | 0.627 |
|  | VMP | 0.724 | 0.321-1.634 | 0.437 |  |
|  | Others | 0.641 | 0.267-1.600 | 0.341 |  |
| Radiotherapy | No |  |  |  | 0.232 |
|  | Yes | 0.319 | 0.044-2.311 | 0.258 |  |
| R-ISS stage | I |  |  |  | 0.042 |
|  | II | 1.055 | 0.438-2.544 | 0.905 |  |
|  | III | 2.083 | 0.833-5.208 | 0.117 |  |
| Hypercalcemia | No |  |  |  | 0.095 |
|  | Yes | 2.049 | 0.868-4.838 | 0.102 |  |
| Renal insufficiency | No |  |  |  | 0.442 |
|  | Yes | 1.237 | 0.719-2.129 | 0.443 |  |
| Anemia | No |  |  |  | 0.248 |
|  | Yes | 1.381 | 0.799-2.388 | 0.248 |  |
| Bone lesions | No |  |  |  | 0.194 |
|  | Yes | 1.573 | 0.790-3.129 | 0.197 |  |
| Extramedullary disease | No |  |  |  | 0.618 |
|  | Yes | 0.846 | 0.436-1.642 | 0.621 |  |
| Unsupervised cluster | A |  |  |  | < 0.001 |
|  | B | 1.074 | 0.593-1.945 | 0.813 |  |
|  | C | 4.081 | 2.005-8.308 | < 0.001 |  |
| Supervised cluster | A |  |  |  | < 0.001 |
|  | B | 1.029 | 0.556-1.905 | 0.928 |  |
|  | C | 4.793 | 2.418-9.498 | < 0.001 |  |
| MTV | Low |  |  |  | < 0.001 |
|  | Moderate | 1.136 | 0.526-2.451 | 0.746 |  |
|  | High | 2.912 | 1.591-5.328 | < 0.001 |  |

Supplementary Table 1. Univariate Cox regression analysis for PFS in subjects without autologous stem cell transplantation

PFS, progression-free survival; VTD, bortezomib, thalidomide, and dexamethasone; VMP, bortezomib, melphalan, and prednisone; R-ISS, Revised Multiple Myeloma International Staging System; MTV, metabolic tumor volume

Supplementary Table 2. Multivariate Cox regression analysis for PFS in subjects without autologous stem cell transplantation

PFS, progression-free survival; MTV, metabolic tumor volume

|  |  | **Unsupervised cluster** | | | **Supervised cluster** | | | **MTV** | | |
| --- | --- | --- | --- | --- | --- | --- | --- | --- | --- | --- |
| **Variable** | **Categories** | **Hazard ratio** | **95% confidence interval** | **P** | **Hazard ratio** | **95% confidence interval** | **P** | **Hazard ratio** | **95% confidence interval** | **P** |
| R-ISS stage | I |  |  |  |  |  |  |  |  |  |
|  | II | 1.032 | 0.420-2.539 | 0.945 | 0.903 | 0.366-2.228 | 0.825 | 1.018 | 0.396-2.620 | 0.970 |
|  | III | 1.655 | 0.630-4.347 | 0.307 | 1.305 | 0.475-3.580 | 0.606 | 1.900 | 0.696-5.186 | 0.210 |
| Unsupervised cluster | A |  |  |  |  |  |  |  |  |  |
|  | B | 1.000 | 0.542-1.846 | 1.000 |  |  |  |  |  |  |
|  | C | 3.360 | 1.585-7.125 | 0.002 |  |  |  |  |  |  |
| Supervised cluster | A |  |  |  |  |  |  |  |  |  |
|  | B |  |  |  | 0.982 | 0.519-1.856 | 0.955 |  |  |  |
|  | C |  |  |  | 4.067 | 1.889-8.757 | < 0.001 |  |  |  |
| MTV | Low |  |  |  |  |  |  |  |  |  |
|  | Moderate |  |  |  |  |  |  | 1.290 | 0.561-2.968 | 0.550 |
|  | High |  |  |  |  |  |  | 2.830 | 1.542-5.194 | < 0.001 |
